# 2.P. Round table: Covid-19 pandemic: the rise or downfall of public health

**DOI:** 10.1093/eurpub/ckac129.118

**Published:** 2022-10-25

**Authors:** Organised by: EUPHA, Chair persons: Iveta Nagyova (EUPHA), Marieke Verschuuren (EUPHA)

## Abstract

This year, we are celebrating 30 years of the European Public Health Association. But celebrating this after over two years of COVID-19 and the Russian reinvasion of Ukraine, invites us all to reflect on the use, misuse and non-use of public health. Over the years, our approach to public health has evolved. At the start of the 20th century, public health emphasized the inequalities created by the environments in which people lived, including housing, sanitation, and nutrition. By the end of the century, this extended to the political and commercial determinants of health and the concept of planetary health, which would later be encapsulated by the 2016 Vienna Declaration on Public Health. Given this comprehensive approach, supported by a much greater body of knowledge produced by many disciplines, public health should have been in the driver's seat when the world was hit with COVID-19. But it was not. Its expertise was often absent from COVID-19 response teams. Policies were often driven by panic in the face of visions of overwhelmed hospitals. Restrictions on mixing were essential until more was known about this new virus but there were failures to appreciate the impact that these measures would have on those already disadvantaged, many in precarious employment in public-facing jobs and overcrowded accommodation. As a consequence, existing health inequalities deepened. It seemed that much that had been learned over the preceding decades had been forgotten. In this roundtable, we seek to explain why, and what needs to change in order to refocus the centrality of public health on supporting and creating fair societies as a prerequisite for health for all.

So, is COVID-19 the downfall or the new rise of public health? In this Round Table, we will discuss the questions as follows.

1. Where was public health during COVID-19?

2. Why were social factors ignored during COVID-19?

3. Why has public health not used/is not using the momentum created by COVID-19?

4. And the main question: How can we create a fair society?

**Key messages:**

• Public health needs to strengthen its core activities.

• Advocacy is a key role for public health.

**Speakers/Panellists:**

**Martin McKee**

LSHTM, London, UK

**Natasha Azzopardi Muscat**

WHO, Msida, Malta

**Dineke Zeegers Paget**

EUPHA

**Klaus D Pluemer**

Independent Public Health & Health Promotion Consultant, Duesseldorf, Germany

**Caroline Costongs**

EuroHealthNet, Brussels, Belgium

